# An evaluation and selection problems of OSS-LMS packages

**DOI:** 10.1186/s40064-016-1828-y

**Published:** 2016-03-01

**Authors:** Belal Najeh Abdullateef, Nur Fazidah Elias, Hazura Mohamed, A. A. Zaidan, B. B. Zaidan

**Affiliations:** Faculty of Information Science and Technology, Universit Kebangsaan Malaysia, Bangi, Malaysia; Faculty of Arts, Computing and Creative Industry, Universiti Pendidikan Sultan Idris, Tanjung Malim, Malaysia; Faculty of Engineering, Multimedia University, Cyberjaya, Malaysia

**Keywords:** Learning management system, Open source software, Software evaluation criteria, Multi-criteria decision making

## Abstract

The evaluation and selection of inappropriate open source software in learning management system (OSS-LMS) packages adversely affect the business processes and functions of an organization. Thus, comprehensive insights into the evaluation and selection of OSS-LMS packages are presented in this paper on the basis of three directions. First, available OSS-LMSs are ascertained from published papers. Second, the criteria for evaluating OSS-LMS packages are specified.according to two aspects: the criteria are identified and established, followed by a crossover between them to highlight the gaps between the evaluation criteria for OSS-LMS packages and the selection problems. Third, the abilities of selection methods that appear fit to solve the problems of OSS-LMS packages based on the multi-criteria evaluation and selection problem are discussed to select the best OSS-LMS packages. Results indicate the following: (1) a list of active OSS-LMS packages; (2) the gaps on the evaluation criteria used for LMS and other problems (consisting of main groups with sub-criteria); (3) use of multi-attribute or multi-criteria decision-making (MADM/MCDM) techniques in the framework of the evaluation and selection of the OSS in education as recommended solutions.

## Background

A learning management system (LMS) is a web-based software application used to organize, implement, and evaluate education. LMS packages provide online learning material, evaluation, and collaborative learning environment. A number of LMSs, such as ATutor, Claroline, and Moodle, have been produced with an open source software license. These free-licensed LMSs are extremely popular for e-learning (Awang and Darus 2011). Open source software (OSS) is software without a license fee and includes its computer program source code. OSS is a means of addressing the rising costs of campus-wide software applications while developing a learner-centered environment (van Rooij 2012; Williams van Rooij 2011).

The number of available OSS in LMSs (OSS-LMSs) online is continuously growing and gaining considerable prominence. This repertoire of open source options is important for any future planner interested in adopting a learning system to evaluate and select existent applications. In response to growing demands, software firms have been producing a variety of software packages that can be customized and tailored to meet the specific requirements of an organization (Jadhav and Sonar 2011; Cavus 2011). The evaluation and selection of inappropriate OSS-LMS packages adversely affect the business processes and functions of the organization. The task of OSS-LMS package evaluation and selection has become increasingly complex because of the (1) difficulties in the selection of appropriate software for business needs given the large number of OSS-LMS packages available on the market, (2) the lack of experience and technical knowledge of the decision maker, and (3) the on-going development in the field of information technology (Lin et al. 2007; Jadhav and Sonar 2009a, b; Cavus 2010).

The task of OSS-LMS evaluation and selection is often assigned under schedule pressure, and evaluators may not have time or experience to plan the evaluation and selection in detail. Therefore, evaluators may not use the most appropriate framework for evaluating and selecting OSS-LMS packages (Jadhav and Sonar 2011). Evaluating and selecting an OSS-LMS package that meets the specific requirements of an organization are complicated and time-consuming decision-making processes (Jadhav and Sonar 2009a, b). Therefore, researchers have been investigating an improved means of evaluating and selecting OSS-LMS packages.

The comprehensive insights into the evaluation and selection of OSS-LMS packages in this paper are based on three directions: available OSS-LMSs from published papers are investigated; the criteria for evaluating OSS-LMS packages are specified; the abilities of the selection methods that appear fit to solve the problem of OSS-LMS packages based on multi-criteria evaluation and selection problem are discussed to select the best OSS-LMS packages. This paper is organized as follows. “Research method” section investigates the method for evaluating and selecting OSS-LMS packages. Section “Conclusions” and 4 discuss the limitations and research contributions of the study, respectively. Section 5 concludes.

## Research method

**Fig. 1:**
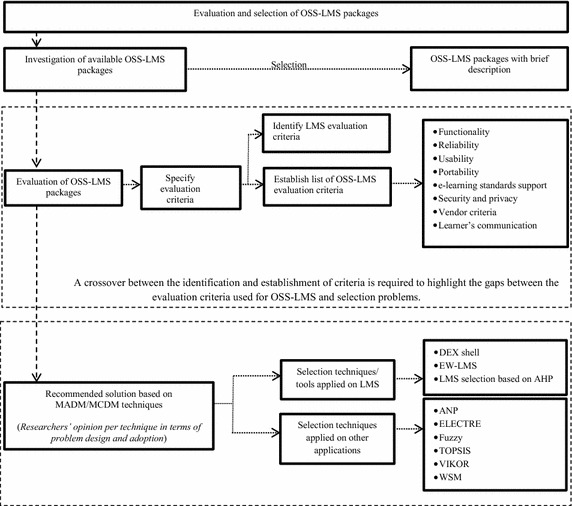
Conceptual framework of the design and contribution of the study

This study is based on the current and active OSS in the education field and presents a list of active OSS-LMS packages, the evaluation criteria with their descriptions to evaluate OSS-LMS packages, and the multi-attribute or multi-criteria decision-making (MADM/MCDM) techniques that are used as recommended solutions to select the best OSS-LMS packages. This study aims to (a) provide a summary of available OSS-LMS reviews and (b) bridge any gaps in technical literature regarding the evaluation and selection of OSS-LMSs by using MADM/MCDM. The conceptual framework (Fig. 1) offers an overview of the research design.

The range of our research in terms of the evaluation and selection of OSS-LMSs based on MADM/MCDM techniques applies only to the LMS packages detected in the search engine databases we used. The review of technical literature was conducted in early 2014 by using three electronic databases: Elsevier’s ScienceDirect, IEEE Xplore, and Web of Science. The search query included the keywords “evaluation learning management system,” “evaluation and selection learning management system,” “e-learning system,” “open source,” and “open source software.” In the process, the title, abstract, conclusion, and methodology were also reviewed to filter the papers by using the scope and inclusion criteria.

This portion describes an analysis of the information research taken through selected published papers. This section consists of three parts: investigation of availability of OSS LMS packages; evaluation criteria classification related to OSS LMS; recommended solutions for software selection problem based on MADM/MCDM techniques.

### Available OSS-LMS packages

We surveyed papers that featured open source LMSs. After analyzing the scope of these papers, 55 studies were selected. From the review, we created an initial taxonomy of 5 categories. We used 4 papers for the adoption of OSS-LMS, 27 papers for the evaluation process, 12 papers for system-based reports, 8 papers for the utilization of an OSS-LMS, and 4 papers for the simple mention of OSS-LMSs (Abdullateef et al. 2015).

This survey aims to identify systems known to decision makers who intend to adopt such systems in their educational institutes. The following table lists OSS-LMSs and the papers in which they are cited.

Table 1 presents the frequency of references. The Moodle system is the most popular OSS-LMS because it is cited in over 40 papers. The Sakai system is the second most popular according to the amount of mentions. The Dot LRN, Claroline, and ATutor systems are fairly equal in the number of references. The Dokeos, Online Learning and Training (OLAT), and LON-CAPA systems are not as popular as the Moodle system and have 9 or less references each. WeBWork, Spaghetti Learning, and Bodington systems are mentioned in only 2 papers. The least cited systems are Totara LMS, Open Source University Support System (OpenUSS), Online Platform for Academic Learning (OPAL), LearnSquare, LogiCampus, Ganesha LMS, eFront, Chamilo, Canvas, and Bazaar LMS. The Moodle system can be deduced as the most studied system because it has the highest amount of references (Table 1).

**Table 1:** OSS-LMSs and frequency in papers

LMS OSS	References
ATutor	Rodríguez Ribón et al. (2013), Graf and List (2005), Cavus (2010), Hailong et al. (2009), Aydin and Tirkes (2010), Skellas and Ioannidis (2011), Bri et al. (2008), Erguzen et al. (2012), Itmazi and Megías (2005), Pecheanu et al. (2011)
Bazaar LMS	Itmazi and Megías (2005)
Bodington	Al-Ajlan et al. (2008), Bri et al. (2008)
Canvas	Kokensparger and Brooks (2013)
Chamilo	Jianxia et al. (2011)
Claroline	Rodríguez Ribón et al. (2013, Cavus (2010), Al-Ajlan et al. (2008), Weinbrenner et al. (2010), Skellas and Ioannidis (2011), Bri et al. (2008), Ligus et al. (2012), Dehnavi and Fard (2011), Erguzen et al. (2012), Itmazi and Megías (2005), Mohd Bekri et al. (2013) Pecheanu et al. (2011)
Dokeos	Graf and List (2005), Al-Ajlan et al. (2008), Aydin and Tirkes (2010), Skellas and Ioannidis (2011), Bri et al. (2008), Ligus et al. (2012), Mohd Bekri et al. (2013), Pecheanu et al. (2011)
eFront	Muhammad et al. (2011)
Ganesha LMS	Itmazi and Megías (2005)
ILIAS	Graf and List (2005), Cavus (2010), Bri et al. (2008), Dehnavi and Fard (2011), Erguzen et al. (2012), Itmazi and Megías (2005), Pecheanu et al. 2011
KEWL	Keats and Beebe (2004), Bri et al. (2008), Stoltenkamp and Kasuto (2011), Itmazi and Megías (2005), Mohd Bekri et al. (2013)
LearnSquare	Mekpiroon et al. (2008)
LRN	Ros et al. (2013), Gil et al. (2008) Graf and List (2005), Caminero et al. (2013), Cavus (2010), Pesquera et al. (2011), Tawfik et al. (2012), Bri et al. (2008), Tawfik et al. (2013), Chu et al. (2012), Erguzen et al. (2012), Itmazi and Megías (2005), Pecheanu et al. (2011)
LogiCampus	Bri et al. (2008)
LON-CAPA	Graf and List (2005), Bri et al. (2008), Itmazi and Megías (2005), Pecheanu et al. (2011)
Moodle	Ros et al. (2013), Rodríguez Ribón et al. (2013), Wen and Lin (2007), Broisin et al. (2005), del Blanco et al. (2012), Graf and List (2005), Caminero et al. (2013), Cavus (2010), Nagi and Suesawaluk (2008), Al-Ajlan et al. (2008), Terbuc (2006), Tick (2009) Arnold and Fisler (2010), Pishva et al. (2010), Aydin and Tirkes (2010), Goyal and Purohit (2010), Weinbrenner et al. (2010), Ai-Lun et al. (2011), Skellas and Ioannidis (2011), Ahmad et al. (2011a, b), Tawfik et al. (2012), Hargis et al. (2012), Schober and Keller (2012), Bri et al. (2008), Tawfik et al. (2013), Ligus et al. (2012), Alier et al. (2010), Capiluppi et al. (2010), Chu et al. (2012), Conde et al. (2014), Corrado et al. (2012), Dehnavi and Fard (2011), Erguzen et al. (2012), Alier et al. (2012), Itmazi and Megías (2005), Romero et al. (2013), Mohd Bekri et al. (2013), Pecheanu et al. (2011), Williams van Rooij (2011), Sarrab and Rehman (2014)
OLAT	Cavus (2010), Al-Ajlan et al. (2008), Arnold and Fisler (2010), Aydin and Tirkes (2010), Skellas and Ioannidis (2011), Schober and Keller (2012), Rossi and Carletti (2011), Mohd Bekri et al. (2013), Pecheanu et al. (2011)
OPAL	Heller et al. (2012)
OpenUSS	Graf and List (2005)
Sakai	Ros et al. (2013), del Blanco et al. (2012), Graf and List (2005), Caminero et al. (2013), Cavus (2010), Al-Ajlan et al. (2008), Hao et al. (2009), Pishva et al. (2010), Wannous and Nakano (2010), Tawfik et al. (2012), Hargis et al. (2012), Bri et al. (2008), Tawfik et al. (2013), Ligus et al. (2012), Chu et al. (2012), Erguzen et al. (2012), Alier et al. (2012), Mohd Bekri et al. (2013), Pecheanu et al. (2011), Williams van Rooij (2011)
Spaghetti learning	Graf and List (2005), Itmazi and Megías (2005)
Totara LMS	Al-Ajlan et al. (2008)
WeBWorK	Gotel et al. (2008), Itmazi and Megías (2005)

Table 2 lists the summaries of OSS-LMS packages. The list consists of 23 OSS-LMS packages, along with their respective websites, and a brief description by the vendor to help administrators or decision makers who intend to evaluate and select an OSS-LMS.

**Table 2:** Summaries of OSS-LMSs

OSS-LMS	Brief description
ATutor	ATutor is a web-based OSS-LMS designed with adaptability and accessibility in mind. The administrators can install or update the system in minutes; the system can also customize templates for a new look and easily extend the functionality with feature modules
	http://www.atutor.ca/
Bazaar LMS	Bazaar is a web-based content hosting platform. This LMS is designed with users and administration in mind. The system is available to the education community as an OSS alternative to proprietary and expensive commercial systems
	https://www.openhub.net/p/5085
Bodington	Bodington is a free OSS-LMS used in colleges and universities worldwide. Bodington is used to support teaching, learning, and researching across a range of learning institutes
	http://elearning-india.com/Learning-Management-System/bodington.html
Canvas	Canvas is freely available under the AGPLv3 license as OSS. This system allows teachers to collaboratively design and transfer curricula for professional e-learning
	https://github.com/instructure/canvas-lms/wiki
Chamilo	Chamilo is project software created in 2001 as OSS in a radical manner. Chamilo aims to provide users a collaboration platform and the best e-learning in the OSS world
	http://www.chamilo.org/en/about-chamilo
Claroline	Claroline is a user-friendly OSS that can easily deploy a dedicated learning and online collaboration platform. This system is available in a number of languages
	http://www.claroline.net/type/claroline
Dokeos	DOKEOS is a complete OSS-LMS created in 1999 at the University of Louvain. The system creates e-learning solutions and trains providers and multinationals with their online training projects
	http://www.dokeos.com/
eFront	eFront LMS offers the best open source solutions through the best of e-learning. The system is flexible, powerful, effective, and fully functional
	http://www.efrontlearning.net/
Ganesha LMS	Ganesha is a free OSS-LMS under the GPL published by Anéma. Ganesha is used to handle course administration and e-learning collaboration. The system also supports several languages: Arabic, English, French, Portuguese, and Spanish
	http://ganesha.fr/index.php?
ILIAS	ILIAS is available as an OSS-LMS under the GNU GPL. This system provides full transparency and no license fees. ILIAS is a powerful system for teaching and learning. This LMS is the first LMS to be SCORM compliant
	http://www.ilias.de/
KEWL	Knowledge Environment for Web-Based Learning (KEWL) is a free OSS-LMS under the GNU GPL. KEWL provides a comprehensive learning management tool for online learning situations
	http://technologysource.org/article/knowledge_environment_for_webbased_learning_(kewl/)
LearnSquare	LearnSquare is a Thai OSS-LMS that supports the e-learning process. This system is compatible with the SCORM standard. The learner can learn at his leisure at any time through the media, papers, pictures, sounds, and videos that can interact with the virtual classroom, which is considered normal for extensive educational opportunities
	http://www.atom.rmutphysics.com/charud/oldnews/0/286/15/6/mechanical/mechanical/index.php-mod=Message&op=aboutus.htm
.LRN	.LRN (dot learn) is a community of educators, designers, and software developers who partner together to drive innovation in the education field. The organizations can save money to develop people skills and curriculum with this free OSS license
	http://dotlrn.org/
LogiCampus	LogiCampus is a free OSS-LMS for course management and distance learning. This system provides a single sign-on for students, staff, and faculty and offers additional features, in addition to a distance learning/course management system
	https://www.openhub.net/p/logicampus
LON-CAPA	LON-CAPA is free a OSS-LMS that supports a full-featured learning content management, course management, and assessment system
	http://www.lon-capa.org/overview.html
Moodle	One of the most popular open source LMSs is Moodle. Moodle is an LMS designed to provide educators, administrators, and learners with a single strong, secure, and integrated system for learning environments
	Moodle.org
OLAT	OLAT is an OSS-LMS tailored to the needs of higher education institutes and universities. OLAT is provided in several languages; the system offers diverse functionality for all the needs in education environments
	http://www.olat.org/
OPAL	OPAL is the central learning platform of Saxonian universities. OPAL is technologically based on OLAT. OPAL is OSS-LMS adjusted to the needs of Saxonian universities.
	https://www.bps-system.de/cms/en/products/opal/
OpenUSS	OpenUSS is an OSS-LMS based on ASP model, which is used in universities, schools, and companies. This system provides users the flexibility to use their selected applications.
	http://openuss.sourceforge.net/openuss/index.html
Sakai Spaghetti learning	Sakai is an OSS-LMS project that provides a flexible and feature-rich environment for teaching, learning, research, and other collaborations. Sakai continually evolves according to the needs of the faculty members, students, and organizations
	https://sakaiproject.org/
	Spaghetti learning is an OSS-LMS written in PHP and is used in numerous universities worldwide. The features of the system include a WYSIWYG editor, trendy graphics and layout, chat and emoticons, storage of learning object and file lessons in logical folders, statistics, and session time and total time in course
	http://www.bigwebmaster.com/2130.html
Totara LMS	Totara is an OSS-LMS designed to meet the learning management needs of enterprises. This system supports several languages: Chinese, English, French, German, Italian, Japanese, Spanish, Portuguese, and Polish
	http://www.totaralms.com/
WeBWorK	WeBWorK is an online homework open source system for science and math courses. WeBWorK is supported by NSF and MAA. This system comes with a national problem library of over 20,000 homework problems. WeBWorK supports the courses in discrete mathematics, probability and statistics, differential equations, single and multivariable calculus, and linear algebra
	http://en.wikipedia.org/wiki/WeBWorK

### Evaluation criteria of OSS-LMS packages

Software packages are evaluated to determine if they are suited to functional, non-functional, and user requirements. By comparing a well-prepared list of criteria, along with a number of realistic analyses, the evaluator can decide if the software is appropriate for his customer (Radwan et al. 2014). According to our study of technical literature, we selected the method for evaluating software on the basis of the following steps:

1. Determine the availability of an OSS-LMS packages from a list of possibly suitable software (Blanc and Korn 1992; Jadhav and Sonar 2009a, b; Cavus 2011; Graf and List 2005).
2. Specify the evaluation criteria for OSS-LMS packages (Jadhav and Sonar 2011; Cavus 2011).

The available OSS-LMS packages are presented in the previous section. The specification of the criteria for software evaluation is explained in detail in the following sections.

#### Specification of the evaluation criteria for OSS-LMS packages

The specification of evaluation criteria for OSS-LMS packages is divided into three main parts, namely, *identified evaluation criteria, established evaluation criteria*, and crossover between the identified and established evaluation criteria.

### Identified evaluation criteria for OSS-LMS packages

 A category was used for the evaluation process of the 27 papers were selected when surveyed the OSS-LMS packages. A total of 16 papers evaluated the learning process, whereas 11 papers evaluated the LMS. We selected the 11 papers, as well as 1 paper from the Google Scholar database that also evaluated OSS-LMS, to obtain the evaluation criteria for OSS-LMS. These studies are described in Table 3. The table offers a brief description about the evaluation criteria and which LMS was used for the evaluation process.

**Table 3:** Brief description about the evaluation criteria and sample of LMS

References	Sample of LMS	Evaluation criteria
Graf and List (2005)	ATutor, Dokeos, dotLRN, ILIAS, LON-CAPA, Moodle, OpenUSS, Sakai, and Spaghetti learning	Learning objects, communication tools, management of user data, usability, technical aspects, adaptation, administration, and course management. Each criterion has sub-criteria
Arh and Blazic (2007), Pipan et al. (2007)	Blackboard, CLIX, and Moodle	Usability testing; student’s learning environment; system, technology, and standards; and tutoring and didactics. Each criterion has sub-criteria
Al-Ajlan et al. (2008)	Desire2Learn, KEWL, ANGEL Learning Management Suite, eCollege, Blackboard, Moodle, Claroline, Dokeos, OLAT, and Sakai	Learner tools (communication, productivity, and student involvement tools), support Tools (administration, course delivery, and content development Tools), technical specifications (hardware/software and pricing/licensing tools). Each criterion has sub-criteria
Bri et al. (2008)	Blackboard, WebCT, Moodle, and Sakai	Upload and share documents, create content online in HTML, online discussions, grade discussions/participation, online chat, student peer review, online quizzes/surveys, online gradebook, student submission of documents, self-assessment of submission, student workgroups, student journals, and embedded glossary
Aydin and Tirkes (2010)	Moodle, ATutor, Dokeos, and OLAT	Support and compatibility to standards (AICC, SCORM), multiple language support, online exam, XML support, chat and group work, ease of installation and maintenance, follow-up of student’s learning process (including content development and content authoring/editing tools, modularity), user authentication, and survey and forum support
Muhammad et al. (2011)	eFront and VULMS	Usability features (feedback/interactivity, learning material, assessment, visibility, learner facilitation and support, error handling and prevention, and collaboration support)
Cavus (2010, 2011)	WebCT, Moodle, and Blackboard	Learner environment, pedagogical factors, instructor tools, course and curriculum design, administrator tools, and technical specification. Each criterion has sub-criteria
Pecheanu et al. (2011)	+CMS, ATutor, Claroline, Dokeos, dotLRN, OpenACS, Drupal, ILIAS, LON-CAPA, Mambo, Moodle, MySource Matrix, OLAT, Plone, and Sakai	Three categories of criteria, build-in applications (tools), technical aspects, and usability. Each criterion has sub-criteria
Srđević et al. (2012)	Blackboard, CLIX, and Moodle	Student’s learning environment; system, technology, and standards category; and tutoring and didactics. Each criterion has sub-criteria
Leba et al. (2013)	Moodle	Pedagogical methods implemented in the system, users security, synchronous interactivity, asynchronous interactivity (forum, chat, e-mail), online accessibility, scale = 200 (number of participants involved simultaneously in a learning activity), ensure the quality of the technical characteristics for the didactical support, symmetry of the system (degree of focusing on each participant), interactivity (response time), system tools available for learning activities, level of cooperation and communication of one student with other students and professors, possibility to integrate information from different sources and represent it in different modes, costs of each participant involved in a learning activity, time (possibility to browse content at own pace), and flexibility of the system to upgrade according to user suggestions

### Established evaluation criteria for OSS-LMS packages

 From our research of technical literature, we combined and classified a collection of criteria suitable for OSS-LMS evaluation. We also defined the meaning of each evaluation criterion. The criteria are categorized into several groups such as functionality, reliability, usability, efficiency, maintainability, and portability. These criteria have been featured in several studies (Franch and Carvallo 2002, 2003; Morisio and Tsoukias 1997; Oh et al. 2003; Ossadnik and Lange 1999; Rincon et al. 2005; Welzel and Hausen 1993; Stamelos et al. 2000; Jadhav and Sonar 2009a, b). Among the ISO/IEC standards related to software quality, ISO/IEC 9126-1 specifically provides a quality model definition, which is used as a framework for software evaluation (Jadhav and Sonar 2009a, b). The rest of the criteria evaluate e-learning standards, security, privacy, vendor criteria, and learner’s communication environment. The efficiency of an LMS is evaluated by the sub-criteria of the usability group. The following subsections explain the criteria groups with their sub-criteria.

#### Functionality group

Functionality is the ability of the software to provide functions that meet the user’s requirements when using the software under specific conditions (Bevan 1999). Functionality is used to measure the level in which an LMS satisfies the functional requirements of an organization (Jadhav and Sonar 2011). The functional group includes several criteria: course development, activity tracking, and assessment. Course development is a web interface for organizing the course’s materials. Activity tracking is important for students. Hence, we focused on the criteria that cover the students’ progress: analysis of current data, time analysis, and sign-in data. Assessment is the possibility for the tutor to test the student through various means (Arh and Blazic 2007). Table 4 presents the criteria with their sub-criteria, along with a description and availability of each sub-criterion.

**Table 4:** Functionality group

References	Criterion	Sub-criterion	Brief description	Procedure mentioned
Al-Ajlan et al. (2008), Arh and Blazic (2007), Aydin and Tirkes (2010), Bri et al. (2008), Cavus (2010, 2011), Graf and List (2005), Pecheanu et al. (2011), Pipan et al. (2007), Srđević et al. (2012)	Course development	Online editor for course organization	The teacher can easily modify the structure of a course and content. The system can automatically generate the content navigation	Yes
		Upload/download of resources packages	HTML pages, images, flash movies, pdf, and MS word files can be up-downloaded in a single Zip archive	Yes
		Linking	The system provides links between content pages, cross-course links, links to student tools	Yes
		Course creation/deletion	The system provides course creation and deletion	No
		Course templates	The system provides different course templates	
Al-Ajlan et al. (2008), Arh and Blazic (2007), Aydin and Tirkes (2010), Bri et al. (2008), Cavus (2010, 2011), Graf and List (2005), Leba et al. (2013), Muhammad et al. (2011), Pecheanu et al. (2011), Pipan et al. (2007), Srđević et al. (2012)	Activity tracking	Activity tracking during the learning process	The system provides activity tracking through the learning process	Yes
		Statistical reports of student progress	The reports aim to provide the teacher a perception of what occurs in a course	Yes
		Participant administration	The system provides participant administration. A course teacher can check the processing status of the course and participants	Yes
		Login analysis	The system provides login analysis. A course teacher can view the time and date of first and last log-ins to a course	Yes
		Time analysis	The system provides time analysis. A course teacher can view the time spent by each student on the course	Yes
		Self-assessing to student	The system provides self-assessment to the student. The student can assess himself using this feature	No
		Online grading	The system provides online grading	No
		Student transcript	The system provides student transcript	No
Al-Ajlan et al. (2008), Arh and Blazic (2007), Aydin and Tirkes (2010), Bri et al. 2008, Graf and List (2005), Muhammad et al. (2011), Pipan et al. (2007), Srđević et al. (2012)	Assessment	Online quiz editor	The system provides a quiz editor. The tutor can easily create/modify the questions and structure of a test	Yes
		Extensible quiz engine	The system provides an extensible quiz engine with automated analysis for the validity of quiz questions	Yes
		Quizzes import	The system has the ability to import quizzes from other tools and systems	Yes

#### Reliability group

Reliability is the ability of the software package to run consistently without crashing under specific conditions (Jadhav and Sonar 2011). Reliability is used to assess the level of fault tolerance for the software package. Furthermore, reliability can be measured by monitoring the number of failures in a given period of execution for a specific task (Bevan 1999). Table 5 depicts the reliability group, its several sub-criteria, and the description and presence of the procedure.

**Table 5:** Reliability group

References	Criterion	Sub-criterion	Brief description	Procedure mentioned
Jadhav and Sonar (2011), Andreou and Tziakouris (2007)	Reliability	Error prone	Error number/crashes per unit of time	Yes
		Correctness	Ratio of successful task	Yes
		Backup and recovery	Capability of the software to support backup and recovery feature	Yes

#### Usability group

Usability establishes how efficient, convenient, and easy a system is for learning (Kiah et al. 2014). The usability group and sub-criterion descriptions are indicated in Table 6 along with the presence of procedure.

**Table 6:** Usability group

References	Criterion	Sub-criterion	Brief description	Procedure mentioned
Jadhav and Sonar (2011), Sung et al. (2007), Seffah et al. (2006), Kiah et al. (2014), Nielsen (1994), Ossadnik and Lange (1999), Heradio et al. (2012)	Usability	Error reporting	Error reporting and messaging ability of the software package	Yes
		User interface	Ease with which the user can use the interface of the software package	Yes
		Learnability	Ease with which the user to learn and operate the package	Yes
Jadhav and Sonar (2011)		User types	Ability of the software package to support beginners, intermediate, advanced users, or a combination of user types	Yes
Seffah et al. (2006), ISO/IEC9241-11 (1998), Kiah et al. (2014)		Efficiency	Time to accomplish tasks or throughput	Yes
		Satisfaction	User comfort and acceptability of use	Yes

#### Maintainability

Maintainability is the ability of the software to be modified. Modifications may include corrections, improvements, or adaptation of the software to changes in the environment, requirement, and functional specifications (Bevan 1999). Maintainability metrics are difficult to measure in a limited experimental setting; they require long-term real-world evaluation. Therefore, we will not consider the maintainability in our study (Kiah et al. 2014).

#### Portability group

Portability is the capability of software to be transferred from one environment to another. (Bevan 1999; Jadhav and Sonar 2011). Table 7 lists the portability criteria and its several sub-criteria along with their definitions and the presence of the procedure.

**Table 7:** Portability group

References	Criterion	Sub-criterion	Brief description	Procedure mentioned
Jadhav and Sonar (2011)	Portability	Middleware standards	Breadth of the middleware standards supported by the software package	Yes
		DBMS standards	Breadth of the DBMS systems supported by the software package	Yes
		Communication standards	Inter-organizational data exchange standards supported by the software package	Yes
		OS compatibility	Package compatibility with the operating systems	Yes

#### E-learning standards group

E-learning standards evaluate learning resources and provide descriptions of the learners’ profiles. E-learning standards are generally developed within the system to ensure interoperability, reusability, and portability, specifically for learning resources (Arh and Blazic 2007). Table 8 indicates the e-learning standards criteria, including several sub-criteria, the description of each sub-criterion, and the procedures.

**Table 8:** E-learning standards group

References	Criterion	Sub-criterion	Brief description	Procedure mentioned
Arh and Blazic (2007), Gil et al. (2008), Kavcic (2011)	E-learning standards	ADL SCORM	Can import a SCORM compliant package	Yes
		IMS QTI	Can import/export IMS question and testing interoperability	Yes
		IMS LIP	Provide IMS learner information package	Yes
		AICC computer managed instruction (CMI)	An automated testing program for verifying conformance with the CMI	Yes

#### Security and privacy group

An overall evaluation of the security of LMS systems is beyond the scope of this research. Evaluating security requires extensive analysis in several aspects; however, we have obtained certain important criteria regarding security and privacy from (Arh and Blazic 2007) and (Jadhav and Sonar 2011). We have used the security and privacy criteria to establish the ability of a system to safeguard personal data and safeguard communication from attacks and danger on a user’s computer, as well as the user’s level of permission (Arh and Blazic 2007). Table 9 depicts the security and privacy criteria, the sub-criteria, and the presence of the procedure.

**Table 9:** Security and privacy group

References	Criterion	Sub-criterion	Brief description	Procedure mentioned
Arh and Blazic (2007)	Security and privacy of an LMS	Authentication	Standard security practices focus on the handling of authentication credentials and subsequent tokens to prevent replay attack	Yes
		Authorization	After the user has been correctly authenticated, authorization mechanisms decide what the user is allowed to do	Yes
		Validation of input	A system that can be used anonymously must be hardened to validate all input from users	Yes
Jadhav and Sonar (2011)		Audit	Products logging and auditing capabilities	Yes
		Data/document encryption	Package support for data/document encryption	Yes

#### Vendor criteria

Vendor criteria are utilized to evaluate the vendor capabilities of software packages. The vendor criteria are important for selecting software because they offer guides for establishing, operating, and customizing software packages (Jadhav and Sonar 2009a, b, 2011; Lee et al. 2013). Table 10 depicts the vendor criteria with a description of each sub-criterion description and presence of the procedure.

**Table 10:** Vendor group

References	Criterion	Sub-criterion	Brief description	Procedure mentioned
Jadhav and Sonar (2009a, b, 2011)	Vendor	User manual	Is there a user manual with indexes, information, and main commands about the software?	Yes
		Tutorial	Is there a tutorial to learn how to use the software?	Yes
		Troubleshooting guide	Is there a troubleshooting guide?	Yes
		Training	Are there training courses to learn the package?	Yes
		Maintenance and upgrading	Availability for maintenance and upgrading of software. The maintenance and upgrading consists of consultancy and demo	Yes
		Communication	Is there communication between user and vendor?	Yes

#### Learner’s communication group

To ensure continuous communication between teachers and students, LMSs require communication tools that use the latest technology. We use learner’s communication criteria to evaluate continuous communication and interaction (Arh and Blazic 2007). Learner’s communication has two types, namely, communication synchronous and communication asynchronous, (Arh and Blazic 2007). Table 11 describes the learner’s communication criteria, its sub-criteria, and the presence of the procedure.

**Table 11:** Learner’s communication group

References	Criterion	Sub-criterion	Brief description	Procedure mentioned
Cavus (2011), Arh and Blazic (2007)	Communication synchronous	Real-time chat room	Is there real-time chat room between students and tutors?	Yes
		Audio/video conferences	Provide audio and/or video conferences	Yes
	Communication asynchronous	Whiteboard	Provides whiteboard	Yes
		Discussion forums	Availability of discussion forums for knowledge exchange	Yes
		File sharing	Provides file sharing service	Yes
		Internal e-mail	Availability of internal e-mail in the learning environment	Yes
		Online journal	Availability of online journal service	Yes

### Crossover between identified and established evaluation criteria for OSS-LMS packages

To determine the gap between the identified and established evaluation criteria, a crossover between the two is required. Table 12 uses x to indicate the criteria used in the papers. For each group of criteria, a percentage of papers that featured that group are provided.

**Table 12:** Crossover between the identified and established evaluation criteria

Groups of Criteria	Criterion	References									
		Graf and List (2005)	Arh and Blazic (2007), Pipan et al. (2007)	Al-Ajlan et al. (2008)	Bri et al. (2008)	Aydin and Tirkes (2010)	Muhammad et al. (2011)	Cavus (2010), Cavus (2011)	Pecheanu et al. (2011)	Srđević et al. (2012)	Leba et al. (2013)
Functional group	Online editor for course organization	x	x	x		x			x	x	x
	Upload/download of resources packages		x		x				x	x	
	Linking		x						x	x	
	Course creation/deletion	x		x	x	x		x			
	Course templates		x	x				x	x	x	
	Activity tracking during the learning process	x	x	x					x	x	
	Statistical reports of student progress	x	x					x	x	x	
	Participant administration	x	x	x						x	
	Login analysis		x						x	x	
	Time analysis		x			x				x	x
	Self-assessing to student	x			x		x	x			
	online grading			x				x			
	Student transcript				x			x	x		
	Online quiz editor	x	x	x	x	x	x			x	
	Extensible quiz engine		x			x				x	
	Quizzes import		x			x				x	
The functional group cited percentage is 41.8 %											
Reliability group	Robustness										
	Correctness										
	Backup and recovery		x			x		x		x	
The reliability group cited percentage is 13.3 %											
Usability group											
	Error reporting						x				
	User interface	x									
	Learnability										
	User types										
	Efficiency		x								
	Satisfaction		x								
The usability group cited percentage is 6.6 %											
Portability group	Middleware standards										
	DBMS standards										
	Communication standards										
	OS compatibility			x				x	x		
The portability group cited percentage is7.5 %											
E-learning standards	ADL SCORM		x		x	x				x	
	IMS		x			x				x	
	AICC CMI		x		x					x	
The e-learning standard cited percentage 33.3 %											
Security and privacy group	Authentication	x	x					x		x	x
	Authorization	x	x			x		x	x	x	x
	Validation of input		x					x		x	
	Audit							x	x		
	Data/document encryption										
The security and privacy group cited percentage is 34 %											
Vendor group	User manual	x						x	x		
	Tutorial										
	Troubleshooting guide										
	Training										
	Maintenance and upgrading										
	Consultancy	x							x		
	Communication										
	Demo										
The vendor group cited percentage is 6.2 %											
Learner’s communication environment group	Real-time chat room	x	x					x	x	x	x
	Audio/video conferences	x	x			x		x		x	
	Whiteboard		x					x		x	
	Discussion forums	x	x		x	x		x	x	x	x
	File sharing				x			x	x		
	Internal e-mail	x	x					x	x	x	x
	Online journal				x			x			
	Wiki/Blog								x		
The learner’s communication environment group cited percentage is 42.5 %											

Table 12 indicates a gap in the OSS-LMS evaluation criteria. The existing software evaluation criteria are insufficient, and establishing new overall quality criteria are needed to evaluate the OSS-LMS packages. The group criteria are insufficient. If we expand the analysis to calculate the criteria by using the percentage from the papers for each group, we find that the proportion of functional group criteria used is 41.8 %, which does not qualify the applicability of the functional group evaluation to a programmed LMS. The same issue is also present in the other groups. The reliability group has 13.3 %, usability group has 6.6 %, portability group has 7.5 %, e-learning standard has 33.3 %, security and privacy group has 34 %, vendor group has 6.2 %, and learner’s communication environment group has 42.5 %.

On the basis of these issues, we deduce that no group has completed the evaluation criteria compared with an established list. In our view, the problem of these percentages can be interpreted in two ways: first, the applicability of this type of criteria in the evaluation process is insufficient; second, this criterion does not meet international software engineering evaluation standards.

Another problem emerged when the software was evaluated by using several criteria (including functionality, reliability, usability, portability, e-learning standards support, security and privacy, vendor criteria, and learner’s communication environment). Each piece of software has several attributes, and each decision maker has different weights for these attributes. Thus, selecting the suitable software to use is difficult. On one hand, users who aim to use one kind of software may prioritize functionality, usability, and user support rather than other features, whereas users who intend to develop this software in actual education environments would probably target different attributes. On the other hand, LMS package selection (in particular, OSS) is an MADM/MCDM problem where each type of software is considered an available alternative for the decision maker. In other words, the MADM/MCDM problem refers to making preference decisions over the available alternatives that are characterized by multiple and usually conflicting attributes (Zaidan et al. 2014). The process of selecting the OSS-LMS packages involves the simultaneous consideration of multiple attributes to rank the available alternatives and select the best one. Thus, the selection process of the OSS-LMS packages can be considered a multi-criteria decision-making problem. Additional details of the fundamental terms of software selection based on multi-criteria analysis will be provided in the following section.

### Recommended solution techniques based on MADM/MCDM

The useful techniques for dealing with MADM/MCDM problems in the real world are defined as recommended solutions in a collective method to help decision makers organize the problems to be solved and conduct the analysis, comparisons, and ranking of the alternatives or multiple platforms. Accordingly, the selection of a suitable alternative is described in previous literature (Jadhav and Sonar 2009a, b); MADM/MCDM methods seem to be suitable for solving the problem of OSS-LMS package selection. The goals of the MADM/MCDM are as follows: (1) help DMs to choose the best alternative, (2) categorize the viable alternatives among a set of available alternatives, and (3) rank the alternatives in decreasing order of performance (Zaidan et al. 2015; Jadhav and Sonar 2009a, b). Each platform has its own multiple criteria that depend on a matrix with—several names: the payoff matrix, evaluation table matrix (ETM), or decision matrix (DM) (Whaiduzzaman et al. 2014). In any MADM/MCDM ranking, the fundamental terms need to be defined, including the DM or the ETM, LMS, and its criteria (Al-Safwani et al. 2014). The ETM that consists of LMS m and n criteria must be created. With the intersection of each LMS and criteria given as *x*_*ij*_, we obtain matrix (*x*_*ij*_)_m*n_:

$$\begin{array}{*{20}c} { \begin{array}{*{20}c}\qquad\qquad\qquad\qquad {\begin{array}{*{20}c} {C_{1} } & {C_{2} } \\ \end{array} } & {\begin{array}{*{20}c} \ldots & {C_{n} } \\ \end{array} } \\ \end{array} } \\ {DM/ETM = \begin{array}{*{20}c} {\begin{array}{*{20}c} {LMS_{1} } \\ {LMS_{2} } \\ \end{array} } \\ {\begin{array}{*{20}c} \vdots \\ {LMS_{m} } \\ \end{array} } \\ \end{array} \left[ {\begin{array}{*{20}c} {\begin{array}{*{20}c} {x_{11} } & {x_{12} } \\ {x_{21} } & {x_{22} } \\ \end{array} } & {\begin{array}{*{20}c} \ldots & {x_{1n} } \\ \ldots & {x_{2n} } \\ \end{array} } \\ {\begin{array}{*{20}c} \vdots & \vdots \\ {x_{m1} } & {x_{m2} } \\ \end{array} } & {\begin{array}{*{20}c} \vdots & \vdots \\ \ldots & {x_{mn} } \\ \end{array} } \\ \end{array} } \right]} \\ \end{array} ,$$

where $LMS_{1} , LMS_{2 } , \ldots , LMS_{m}$ are possible alternatives that decision makers must score (i.e., Moodle platform); $C_{1} , C_{2 } , \ldots , C_{n}$ are the criteria for measuring each LMS’s performance (i.e., functionality criteria, reliability iteria, usability, etc.). Finally, *x*_*ij*_ is the rating of alternative *LMS*_*i*_ with respect to criterion $C_{j}$. Certain processes need to be conducted to rank alternatives, such as normalization, maximization indicator, adding the weights, and other processes, depending on the method.

For example: Suppose D is the DM to rank the performance of alternative A_i_ (i = {1, 2, 3 and 4}) on the basis of Cj (j = {1, 2, 3, 4, 5 and 6}). Table 13 is an example of a multi-criteria problem reported in (Hwang and Yoon 1981).

**Table 13:** Example of a multi-criteria problem

*A*_*i*_ *C*_*j*_	*C*_*1*_	*C*_*2*_	*C*_*3*_	*C*_*4*_	*C*_*5*_	*C*_*6*_
A1	2	1500	20,000	5.5	5	9
A2	2.5	2700	18,000	6.5	3	5
A3	1.8	2000	21,000	4.5	7	7
A4	2.2	1800	20,000	5	5	5

**Fig. 2:**
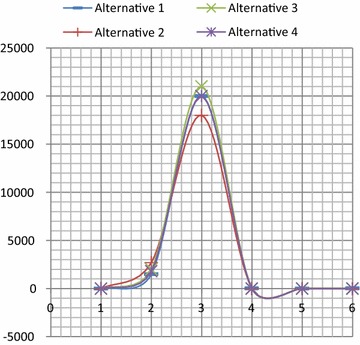
Graphic presentation of the Example in Table 13

The data in the chart is difficult to evaluate because of the large numbers of c2 and c3. See Fig. 2.

**Fig. 3:**
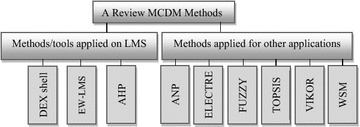
A review MCDM methods

Selecting the best software process from the software on offer is an important aspect of managing an information system. The selection process can be considered a MADM/MCDM problem that can address different and inconsistent criteria to select between predetermined decision alternatives (Oztaysi 2014). We will divide this section into two subsections. The first subsection describes the current selection methods applied for LMS selection. The second examines recent studies related to MCDM techniques applied for other applications, as shown in Fig. 3.

### Selection techniques/tools applied on LMS

MADM/MCDM is an effective approach for addressing various types of decision-making problems. In the field of education, some papers employ MADM/MCDM techniques and tools to evaluate and select the best LMS. These techniques and tools include the decision expert shell (DEX shell) system (Arh and Blazic 2007; Pipan et al. 2007), easy way to evaluate LMS (EW-LMS) (Cavus 2011), and analytic hierarchy process (Srđević et al. 2012). Table 14 presents a brief description of these references, as well as the MADM/MCDM techniques and tools used for selecting the best LMS.

**Table 14:** MADM/MCDM techniques are used to select LMS

References	Selection method technique	Brief description
Arh and Blazic (2007), Pipan et al. (2007) and Bohanec and Rajkovič (1990)	DEX shell system	Provides tools for building and verifying a knowledge base. The system uses the MCDM method
Cavus (2010, 2011)	EW-LMS system	The system utilizes fuzzy value and linear weighted attribute model. The system adopts the MCDM method
Srđević et al. (2012)	AHP	AHP is used to solve an MCDM problem

#### DEX shell system

In this section (Arh and Blazic 2007; Pipan et al. 2007), we looked at the DEX shell system for scoring, ranking, and selecting the best LMS. DEX is developed as an interactive expert system shell that offers tools to create and verify a knowledge base, evaluate choices, and explain the final results. The structure of the knowledge base and evaluation procedures closely match the multi-criteria decision-making paradigm; however, the system considers the consistency of the decision-making process and the weighed sum of the criteria is achieved with limited theoretical justification (Srđević et al. 2012).

#### EW-LMS

Cavus (2011) developed the EW-LMS. This system is a web-based system that can be used easily on the Internet anywhere and anytime. This system is designed as a decision support system that uses a smart algorithm derived from artificial intelligent concepts with fuzzy values. This system adopts fuzzy logic values to assign the weight of each criterion and utilizes the linear weighted attribute model to select the best alternative. However, the technique used to assign the criterion weight is inaccurate because the user weighs in the group arbitrarily uses fuzzy logic values (Jadhav and Sonar 2009a, b).

#### LMS selection based on AHP

Srđević et al. (2012) presented an evaluation method for selecting the most appropriate LMS. The authors propose a breakdown of complex criteria into easily comprehended sub-criteria through a method called analytic hierarchy process (AHP). AHP ranks alternative software when the features are considered and modified and deletes unsuitable software from the evaluation process (Zaidan et al. 2014; Jadhav and Sonar 2009a, b).

AHP was devised by Saaty in 1980. AHP has become a commonly used and widely distributed technique for MCDM. AHP allows the use of both qualitative and quantitative criteria at the same time. It also allows the utilization of independent variables and compares attributes in a hierarchal structure.

In a tree structure, the hierarchy begins at the top and comes down toward the goal. The lower levels correspond to the criteria, sub-criteria, and so on. In this hierarchal tree, the process starts from the leaf nodes and progresses up to the top level. Each output level represents the hierarchy that corresponds to the weight or influence of different branches originating from that level. Finally, the different branches are compared to select the most appropriate alternative on the basis of the attributes (Whaiduzzaman et al. 2014; San Cristóbal 2011; Zaidan et al. 2014; Oztaysi 2014; Srđević et al. 2012; Jadhav and Sonar 2011; Ngai and Chan 2005; Krylovas et al. 2014).

- Step 1: Pairwise comparison between criteria;
- Step 2: Raising the attained matrix to an arbitrarily large power;
- Step 3: Normalizing row sums of the raised matrix using the following equation:

1 $${\text{w}}_{\text{i}} = \frac{{{\text{w}}_{\text{i}}^{ '} }}{{\mathop \sum \nolimits_{{{\text{j}} = 1}}^{\text{m}} {\text{w}}_{\text{i}}^{ '} }}$$

where

$$w_{i}^{'} = \mathop \sum \limits_{j = 1}^{m} a_{ij}^{'} i,\quad j = 1,2, \ldots ,m$$

and $a_{ij}^{\prime}$ is the corresponding element of *i*^th^ and *j*^th^ criterion for the raised matrix.

- Step 4: Rating the alternatives in terms of the criteria;
- Step 5: Synthesizing the vectors from the last two steps to obtain the final priority vectors for the alternatives

### Selection techniques applied for other applications

Decision-making theories have been applied successfully in different fields over the past few decades. The variety and diversity of MADM/MCDM applications have helped decision makers. MADM/MCDM can allow the application of multiple conflicting criteria. One main objective of this study is to introduce a critical assessment of available MADM/MCDM approaches and describe how these approaches are used in OSS-LMS selection (Wang et al. 2013). We selected recent studies related to decision-making selection techniques, which are listed as follows according to Refs. (Wang et al. 2013; Jadhav and Sonar 2011; Triantaphyllou 2000; Triantaphyllou et al. 1998; Al-Safwani et al. 2014).

#### Analytic network process (ANP)

ANP is defined as a mathematical theory that can handle all types of dependencies systematically. It can be used in numerous fields. ANP was developed by Saaty (Wu and Lee 2007), and includes a multi-criteria decision-making method that compares different alternatives to select the best alternative.

ANP technique allows the addition of an extra relevant criterion to an existing one, which are either tangible or intangible, thus significantly influencing the decision-making process. Furthermore, ANP considers interdependencies for different levels of set criteria. Finally, ANP permits quantitative and qualitative feature analysis, thus making ANP a preferred technique in many real-world situations (Yazgan et al. 2009). ANP is composed of four major steps:

- Step1: Model construction and problem structuring
- Step2: Pairwise comparison matrices and priority vectors
- Step3: Super matrix formation
- Step4: Selection of the best alternatives

#### Elimination and choice expressing reality

Roy and his colleagues at the SEMA consultancy company developed Elimination and Choice Expressing Reality (ELECTRE) in 1991. Since then, several variations of the method have been coined, such as ELECTRE I, ELECTRE II, ELECTRE III, ELECTRE IV, ELECTRE IS, and ELECTRE TRI (ELECTRE Tree). All of these variations consist of two sets of parameters: veto thresholds and the importance coefficient (Mohammadshahi 2013; Whaiduzzaman et al. 2014).

The method is classified as an outranking MCDM method. Compared with previous methods, this approach is computationally complex because the simplest method of ELECTRE was reported to involve up to 10 steps. The mechanics of the method allow it to compare alternatives to determine their outranking relationships. The relationships are utilized to define and/or eliminate the alternatives subdued by others, thus subsequently reducing the amount of available alternatives.

Another feature of ELECTRE is its ability to handle both qualitative and quantitative criteria. This provides a basis for a complete order of different options. The preferred alternatives are weighed against dependence on concordance indices, and their thresholds allow the drafting of graphs that can be later used to obtain the ranking of alternatives (Rehman et al. 2012; San Cristóbal 2011; Whaiduzzaman et al. 2014).

The IF ELECTRE method includes eight steps (Wu and Chen 2009):

#### Step 1: Determine the DM

$${\text{Let}}\;X_{ij} = \left( {\mu_{ij} , \, \nu_{ij} , \, \pi_{ij} } \right),$$

μ_ij_ is the degree of membership of the *i*th alternative with respect to the *j*th attribute, ν _ij_ is the degree of non-membership of the *i*th alternative with respect to the *j*th attribute, and π _ij_ is the intuitionistic index of the *i*th alternative with respect to the *j*th attribute. *M* is an intuitionistic fuzzy DM where

2 $$0 \le \mu_{ij} + \nu_{ij} \le 1,\quad i \, = 1,2, \ldots ,m, \;\; j \, = 1,2, \ldots ,n$$

3 $$\pi_{ij} = 1 - \mu_{ij} - \nu_{ij}$$

$$M = \begin{array}{*{20}c} {A_{1} } \\ \vdots \\ {A_{m} } \\ \end{array} \left[ {\begin{array}{*{20}c} {x_{11} } & \cdots & {x_{1n} } \\ \vdots & \ddots & \vdots \\ {x_{m1} } & \cdots & {x_{mn} } \\ \end{array} } \right]$$

In the DM M, we have m alternatives (from A_1_ to A_m_) and *n* attributes (from x_1_ to x_n_). The subjective importance of attributes, W, is given by the decision maker(s).

#### Step2: Determine the concordance and discordance sets

The method uses the concept of IFS relation to identify (determine) the concordance and discordance set.

The strong concordance set $C_{\text{kl}}$ of $A_{\text{k}}$ and $A_{\text{l}}$ is composed of all criteria where $A_{\text{k}}$ is preferred to $A_{\text{l}}$. In other words, the strong concordance set $C_{\text{kl}}$ can be formulate as

4 $$C_{kl} = \left\{ {j|\mu_{kj} \ge \mu_{lj} ,v_{kj} < v_{lj} \quad and\quad \pi_{kj} < \pi_{lj} } \right\}$$

The moderate concordance set $C_{\text{kl}}^{{''}}$ is defined as

5 $$C_{kl}^{'} = \left\{ {j|\mu_{kj} \ge \mu_{lj} ,v_{kj} < v_{lj} \quad and \quad \pi_{kj} \ge \pi_{lj} } \right\}$$

The weak concordance set $C_{\text{kl}}^{{''}}$ is defined as

6 $$C_{kl}^{''} = \left\{ {j|\mu_{kj} \ge \mu_{lj} \quad and\quad v_{kj} \ge v_{lj} } \right\}$$

The strong discordance set $D_{\text{kl}}$ is composed of all criteria where $A_{\text{k}}$. is not preferred to *A*_1_. The strong discordance set $D_{\text{kl}}$ can be formulated as

7 $$D_{kl} = \left\{ {j |\mu_{kj} < \mu_{lj} ,v_{kj} \ge v_{lj} \quad and\quad \pi_{kj} \ge \pi_{lj} } \right\}$$

The moderate discordance set $$D_{kl}^{{\prime }}$$$${\prime }$$ is defined as

8 $$D_{kl}^{'} = \left\{ {j |\mu_{kj} < \mu_{lj} ,v_{kj} \ge v_{lj} \quad and\quad \pi_{kj} < \pi_{lj} } \right\}$$

The weak discordance set $D_{kl}^{{''}}$ is defined as

9 $$D_{kl}^{''} = \left\{ {j |\mu_{kj} < \mu_{lj} \quad and \quad v_{kj} < v_{lj} } \right\}$$

The decision maker(s) provides the weight in different sets.

#### Step 3: Calculate the concordance matrix

The relative value of the concordance sets is measured by using the concordance index. The concordance index is equal to the sum of the weights associated with these criteria and relations that are contained in the concordance sets.

Thus, concordance index *c*_*kl*_ c between *A*_*k*_ and *A*_*l*_ is defined as follows:

10 $$c_{kl} = w_{c} \times \mathop \sum \limits_{{j \in C_{kl} }} w_{j} + w_{c'} \times \mathop \sum \limits_{{j \in C'_{kl} }} w_{j} + w_{c''} \times \mathop \sum \limits_{{j \in C''_{kl} }} w_{j}$$

where w_C_, w_C’_, w_C’’_ are the weight in different sets defined in Step 2, and w_j_ is the weight of attributes identified in Step 1.

#### Step 4: Calculate the discordance matrix

Discordance index d_kl_ is defined as follows:

11 $$d_{kl} = \frac{{{}_{{j \in D_{kl} }}^{\hbox{max} } w_{D} * \times dis\left( {x_{kj} , x_{lj} } \right)}}{{{}_{j \in J}^{\hbox{max} } dis\left( {x_{kj} , x_{lj} } \right)}}$$

12 $$dis\left( {x_{kj} , x_{lj} } \right) = \sqrt {\frac{1}{2}((\mu_{kj} - \mu_{lj} )^{2} + (v_{kj} - v_{lj} )^{2} + \left( {\pi_{kj} - \pi_{lj} )^{2} } \right)}$$

where w_D*_ is equal to w_D_ or w_D’_ or w_D’’_, which depends on the different types of discordance sets defined in Step 2.

#### Step 5: Determine the concordance dominance matrix

This matrix can be calculated by adopting a threshold value for the concordance index. *A*_*k*_ can only dominate *A*_*l*_ if its corresponding concordance index *c*_*kl*_ exceeds a certain threshold value c^−^, i.e., *c*_*kl*_ ≥ c^−^, and

13 $$c^{ - } = \frac{{\mathop \sum \nolimits_{k = l,k \ne l}^{m} \mathop \sum \nolimits_{l = l,l \ne k}^{m} c_{kl} }}{{m \times \left( {m - 1} \right)}}$$

On the basis of the threshold value, a Boolean matrix *F* can be constructed; the elements of which are defined as

$$f_{kl} = 1,\quad if \; c_{kl} \ge c^{ - } ;\quad f_{kl} = 0, \quad if \; c_{kl} < c^{ - } .$$

Each element of “1” on matrix *F* represents a dominance of one alternative with respect to another.

#### Step 6: Determine the discordance dominance matrix

This matrix is constructed analogously to the *F* matrix on the basis of a threshold value *d*^−^ to the discordance indices. The elements of *g*_*kl*_ of the discordance dominance matrix G are calculated as follows:

14 $$d^{ - } = \frac{{\mathop \sum \nolimits_{k = l,k \ne l}^{m} \mathop \sum \nolimits_{l = l,l \ne k}^{m} d_{kl} }}{{m \times \left( {m - 1} \right)}}$$

$$g_{kl} = 1,\quad if \; d_{kl} \le d^{ - } ; g_{kl} = 0, \quad if\; d_{kl} > d^{ - }$$

The unit elements in the G matrix also represent the dominance relationships between any two alternatives.

#### Step 7: Determine the aggregate dominance matrix

This step involves the calculation of the intersection of the concordance dominance matrix *F* and discordance dominance matrix *G*. The resulting matrix, which is called the aggregate dominance matrix *E*, is defined by using its typical elements *e*_*kl*_ as follows:

15 $$e_{kl} = f_{kl} .g_{kl}$$

#### Step 8: Eliminate the less favorable alternatives

The aggregate dominance matrix E provides the partial-preference ordering of the alternatives. If *e*_*kl*_ = 1, *A*_*k*_ is preferred to *A*_*l*_ for both the concordance and discordance criteria. However, *A*_*k*_ still has the chance of being dominated by other alternatives. Hence, when *A*_*k*_ is not dominated by ELECTRE, the following is obtained:

$$e_{kl} = 1,\;{\text{for}}\;{\text{at}}\;{\text{least}}\;{\text{one}}\;l,\quad l = 1,2, \ldots ,m,\;k \ne l;$$

$$\left( {e_{ik} = 0,\;{\text{for}}\;{\text{all}}\;i,\quad i = 1,2, \ldots ,m,\;i \ne l,\;i \ne k;} \right)$$

This condition appears difficult to apply. However, the dominated alternatives can be easily identified in the *E* matrix. If any column of the *E* matrix has at least one element, this column is “ELECTREcally” dominated by the corresponding row(s). Hence, we simply eliminate any column(s) with an element of one.

#### Fuzzy

Fuzzy theory was introduced by Zadeh in 1965. It is an extensive theory applied to man’s uncertainties when making a judgment. The theory can also rectify doubts associated with available data and information in multiple criteria decision making.

An MCDM model based on fuzzy theory can be used to evaluate and choose a specific alternative that matches the criteria set by the decision maker from a pool of options. Linguistic values represented by fuzzy numbers label suitable replacements and weigh them against importance. A comparison is then conducted between the numerical values and weighed values to determine the true values with Boolean logic and replace them with intervals in the decision-making process (Alabool and Mahmood 2013; Whaiduzzaman et al. 2014).

Let X be the universe of discourse, and X = {x_1_, x_2_,…, x_n_}. A* is a fuzzy set of X that represents a set of ordered couples {(x_1_, µ_A*_(x_1_)), (x_2_, µ_A*_ (x_2_)),…, (x_n_, µ_A*_ (x_n_))}, µ_A*_:X → [0,1] is the function of membership grade “Membership Function” of A*, and µ_A*_ (x_i_) stands for the membership degree of x_i_ in A*.

A fuzzy number represents a fuzzy subset in the universe of discourse X that is both convex and normal. Triangular fuzzy number, trapezoidal fuzzy number, and bell-shaped fuzzy number are types of membership functions. However, this study aims to adopt the triangular fuzzy number type. A triangular fuzzy number is a fuzzy number represented by three points (p1, p2, p3) and (p1 < p2 < p3). The interpreted membership function µ_A*_ of the fuzzy number A* is:

$$\mu A*\left( {\text{x}} \right) = \left\{ {\begin{array}{*{20}l} {0,} \hfill & {X < p1} \hfill \\ {\frac{x - p1}{p2 - p1},} \hfill & {P1 \le x \le p2} \hfill \\ {0, } \hfill & {x \le p3} \hfill \\ \end{array} } \right.$$

#### Technique for order of preferences by similarity to ideal solution

The Technique for Order of Preferences by Similarity to Ideal Solution (TOPSIS) presents a preference index of similarity for ideal solutions. Thus, this approach can reach the closest possible solution to the ideal one and drive the solution as far away as possible from the anti-ideal solution at the same time.

A DM is first needed for this technique. The matrix is normalized using vectors, followed by the identification of both the anti-ideal and ideal solutions defined within the normalized DM. The technique was invented by Hwang and Yoon in 1981. It chooses the alternatives with the shortest and most positive distance from the ideal solution and the most negative distance from the anti-ideal solution. This technique is adopted to select a solution from a set of finite options.

Ideally, the optimal solution has the shortest distance from the ideal solution and the farthest possible distance from the anti-ideal solution at the same time. The cumulative function produced by the TOPSIS technique builds up the distance to be as near as possible from the ideal solution and the opposite from the anti-ideal solution; However, a reference point must be set near the ideal solution (ur Rehman et al. 2012; San Cristóbal 2011; Whaiduzzaman et al. 2014; Oztaysi 2014; Cui-yun et al. 2009). The TOPSIS method includes the following steps:

#### Step 1: Construct the normalized DM

This process tries to transform the various attribute dimensions into non-dimensional attributes. This process allows a comparison across attributes. The matrix $\left( {{\text{x}}_{\text{ij}} } \right)_{\text{m*n}}$ is then normalized from $\left( {{\text{x}}_{\text{ij}} } \right)_{\text{m*n}}$ to matrix ${\text{R}} = \left( {{\text{r}}_{\text{ij}} } \right)_{\text{m*n}}$ by using the normalization method:

16 $$\varvec{r}_{{\varvec{ij}}} = \varvec{x}_{{\varvec{ij}}} /\sqrt {\mathop \sum \limits_{{\varvec{i} = 1}}^{\varvec{m}} \varvec{x}_{{\varvec{ij}}}^{2} }$$

This process will result in a new Matrix R:

17 $$\varvec{R} = \left[ {\begin{array}{*{20}c} {\begin{array}{*{20}c} {\varvec{r}_{11} } & {\varvec{r}_{12} } \\ {\varvec{r}_{21} } & {\varvec{r}_{22} } \\ \end{array} } & {\begin{array}{*{20}c} \ldots & {\varvec{r}_{{1\varvec{n}}} } \\ \ldots & {\varvec{r}_{{2\varvec{n}}} } \\ \end{array} } \\ {\begin{array}{*{20}c} \vdots & \vdots \\ {\varvec{r}_{{\varvec{m}1}} } & {\varvec{r}_{{\varvec{m}2}} } \\ \end{array} } & {\begin{array}{*{20}c} \vdots & \vdots \\ \ldots & {\varvec{r}_{{\varvec{mn}}} } \\ \end{array} } \\ \end{array} } \right]\varvec{ }$$

#### Step 2: Construct the weighted normalized DM

In this process, a set of weights $w = w_{1} , w_{2} , w_{3 } , \cdots ,w_{j} , \cdots , w_{n}$ from the decision maker is accommodated to the normalized DM. The resulting matrix can be calculated by multiplying each column from the normalized DM (R) with its associated weight $w_{j}$. It should be noted that the set of the weights is equal to one

18 $$\mathop \sum \limits_{{\varvec{j} = 1}}^{\varvec{m}} \varvec{w}_{\varvec{j}} = 1$$

This process will result in a new Matrix V:

19 $${\mathbf{V}} = \left[ {\begin{array}{*{20}c} {\begin{array}{*{20}c} {\varvec{v}_{11} } & {\varvec{v}_{12} } \\ {\varvec{v}_{21} } & {\varvec{v}_{22} } \\ \end{array} } & {\begin{array}{*{20}c} \ldots & {\varvec{v}_{{1\varvec{n}}} } \\ \ldots & {\varvec{v}_{{2\varvec{n}}} } \\ \end{array} } \\ {\begin{array}{*{20}c} \vdots & \vdots \\ {\varvec{v}_{{\varvec{m}1}} } & {\varvec{v}_{{\varvec{m}2}} } \\ \end{array} } & {\begin{array}{*{20}c} \vdots & \vdots \\ \ldots & {\varvec{v}_{{\varvec{mn}}} } \\ \end{array} } \\ \end{array} } \right] = \left[ {\begin{array}{*{20}c} {\begin{array}{*{20}c} {\varvec{w}_{1} \varvec{r}_{11} } & {\varvec{w}_{2} \varvec{r}_{12} } \\ {\varvec{w}_{1} \varvec{r}_{21} } & {\varvec{w}_{2} \varvec{r}_{22} } \\ \end{array} } & {\begin{array}{*{20}c} \ldots & {\varvec{w}_{\varvec{n}} \varvec{r}_{{1\varvec{n}}} } \\ \ldots & {\varvec{w}_{\varvec{n}} \varvec{r}_{{2\varvec{n}}} } \\ \end{array} } \\ {\begin{array}{*{20}c} \vdots & \vdots \\ {\varvec{w}_{1} \varvec{r}_{{\varvec{m}1}} } & {\varvec{w}_{2} \varvec{r}_{{\varvec{m}2}} } \\ \end{array} } & {\begin{array}{*{20}c} \vdots & \vdots \\ \ldots & {\varvec{w}_{\varvec{n}} \varvec{r}_{{\varvec{mn}}} } \\ \end{array} } \\ \end{array} } \right]$$

#### Step 3: Determining the ideal and negative ideal solutions

In this process, two artificial alternatives A^*^ (ideal alternative) and *A*^−^ (negative ideal alternative) are defined as follows:

20 $$\begin{aligned} \varvec{A}^{*} & = \left\{ {\left( {\left( {\mathop {{\mathbf{max}}}\limits_{i} \varvec{v}_{ij} |\varvec{j} \in \varvec{J}} \right), \left( {\mathop {{\mathbf{min}}}\limits_{i} \varvec{v}_{ij} |\varvec{j} \in \varvec{J}^{ - } } \right)|\varvec{i} = 1,2, \ldots ,\varvec{m}} \right)} \right\} \\ & \quad = \left\{ {v_{1}^{*} , v_{2}^{*} , \ldots , v_{j}^{*} , \ldots v_{n}^{*} } \right\} \\ \end{aligned}$$

21 $$\begin{aligned} \varvec{A}^{ - } & = \left\{ {\left( {\left( {\mathop {\varvec{m}{\mathbf{in}}}\limits_{\varvec{i}} \varvec{v}_{{\varvec{ij}}} |\varvec{j} \in \varvec{J}} \right), \left( {\mathop {\hbox{max} }\limits_{\varvec{i}} \varvec{v}_{{\varvec{ij}}} |\varvec{j} \in \varvec{J}^{ - } } \right) |\varvec{i} = 1,2, \ldots ,\varvec{m}} \right)} \right\} \\ & \quad = \left\{ {\varvec{v}_{1}^{ - } ,\varvec{ v}_{2}^{ - } , \ldots ,\varvec{ v}_{\varvec{j}}^{ - } , \ldots \varvec{v}_{\varvec{n}}^{ - } } \right\} \\ \end{aligned}$$

*J* is a subset of $\left\{ {i = 1,2, \ldots ,m} \right\}$, which presents the benefit attribute, whereas *J*^−^ is the complement set of *J* and can be noted as $J^{c}$, which is the set of cost attribute.

#### Step 4: Separation measurement calculation based on the Euclidean distance

In the process, the separation measurement is conducted by calculating the distance between each alternative in *V* and ideal vector A^*^ by using the Euclidean distance, which is expressed as follows:

22 $$\varvec{S}_{{\varvec{i}^{*} }} = \sqrt {\mathop \sum \limits_{{\varvec{j} = 1}}^{\varvec{n}} \left( {\varvec{v}_{{\varvec{ij}}} - \varvec{v}_{\varvec{j}}^{*} } \right)^{2} } , \quad \varvec{i} = \left( {1,2, \ldots \varvec{m}} \right)$$

Similarly, the separation measurement for each alternative in *V* from the negative ideal *A*^−^ is given by the following:

23 $$\varvec{S}_{{\varvec{i}^{ - } }} = \sqrt {\mathop \sum \limits_{{\varvec{j} = 1}}^{\varvec{n}} \left( {\varvec{v}_{{\varvec{ij}}} - \varvec{v}_{\varvec{j}}^{ - } } \right)^{2} } , \quad \varvec{i} = \left( {1,2, \ldots \varvec{m}} \right)$$

In the end of Step 4, two values, namely, $S_{{i^{*} }}$ and $S_{{i^{ - } }}$, for each alternative were counted. The two values represent the distance between each alternative and both alternative (the ideal and negative ideal).

#### Step 5: Closeness to the ideal solution calculation

In the process, the closeness of *A*_*i*_ to ideal solution A^*^ is defined as follows:

24 $$\varvec{C}_{{\varvec{i}^{*} }} = \varvec{S}_{{\varvec{i}^{ - } }} /\left( {\varvec{S}_{{\varvec{i}^{ - } }} + \varvec{S}_{{\varvec{i}^{*} }} } \right), \quad 0 < \varvec{C}_{{\varvec{i}^{*} }} < 1, \; \varvec{i} = \left( {1,2, \ldots \varvec{m}} \right)$$

Evidently, $C_{{i^{*} }} = 1$ if and only if (*A*_*i*_ = A^*^). Similarly, $C_{{i^{*} }} = 0$ if and only if (*A*_*i*_ = *A*^−^)

#### Step 6: Ranking the alternative according to the closeness to the ideal solution

The set of alternative $A_{i}$ can now be ranked according to the descending order of $C_{{i^{*} }}$. The alternative with the highest value will have the highest performance.

#### VIKOR

The compromise ranking method, which is also known as VIKOR, is an effective technique with more than one criterion set for decision making. The acronym is derived from “Vise Kriterijumska Optimizacija I Kompromisno Resenje.” The multi-criteria ranking index is developed on the basis of the measurements of proximity to the ideal solution (usually in the form of distance). This technique was introduced by Opricovic in 2004 to optimize the evaluation dynamic and complicated processes through compromising. The technique uses linear normalization; however, the values are not dependent on just criterion evaluation. VIKOR also uses an aggregate function to balance the distance between both the ideal solution and its opposite. This helps the decision maker choose from a set of conflicting solutions (Alabool and Mahmood 2013; Whaiduzzaman et al. 2014; San Cristóbal 2011).

The VIKOR steps are as follows:

#### Step 1: Calculate *x*_i_^*^ and x_i_^−^

25 $$x_{i}^{*} = { \hbox{max} }[\left( {x_{ij} } \right)|j = 1,2, \ldots ,m]$$

26 $$x_{i}^{ - } = { \hbox{min} }[\left( {x_{ij} } \right)|j = 1,2, \ldots ,m]$$

where *x*_*ij*_ is the value of the *i*th criterion function for alternative *x*_*i*_.

#### Step 2: Compute the values of S_j_ and R_j_

27 $$s_{i} = \mathop \sum \limits_{i = 1}^{n} w_{i} \frac{{x_{i}^{*} - x_{ij}^{{}} }}{{x_{i}^{*} - x_{i}^{ - } }}$$

28 $$R_{j} = \hbox{max} \left[ {w_{i} \left( {\frac{{x_{i}^{*} - x_{ij} }}{{x_{i}^{*} - x_{i}^{ - } }}} \right)} \right]\quad i = 1,2, \ldots ,n$$

where *s*_*i*_ and *R*_*j*_ denote the utility measure and regret measure for alternative *x*_*j*_. Furthermore, *w*_*i*_ is the weight of each criterion.

#### Step 3: Compute the values of S^*^, R^*^

29 $$S^{*} = \hbox{min} \left( {S_{j} } \right), \quad S^{ - } = \hbox{max} \left( {S_{j} } \right), \quad j = 1,2, \ldots ,m$$

30 $$R^{*} = \hbox{min} \left( {R_{j} } \right), \quad R^{ - } = \hbox{max} \left( {R_{j} } \right), \quad j = 1,2, \ldots ,m$$

#### Step 4: Determine the value of $Q_{j} for j = 1,2, \ldots ,m$ and rank the alternatives by values of *Q*_*j*_

31 $$Q_{j} = v\left( {\frac{{S_{j} - S^{*} }}{{S^{ - } - S^{*} }}} \right) + \left( {1 - v} \right)\left( {\frac{{R_{j} - R^{*} }}{{R^{ - } - R^{*} }}} \right),$$

where *v* is the weight to maximize group utility and (1 − *v*) is the weight of the individual regret. Usually, v = 0.5; when *v* > 0.5, the index of *Q*_*i*_ will tend to show majority agreement. When *v* < 0.5, the index of *Q*_*i*_ will indicate a dominantly negative attitude.

#### Weighted scoring method

The weighted scoring method (WSM) is a technique used to evaluate and select software packages. Ease of use is the main advantage of this technique. Suppose m alternatives A1, A2,…, Am has n criteria C1, C2,…, Cn.

The alternatives are fully characterized by DM Sij. Suppose that weights W1, W2,…, Wk is the importance value of the criteria. The suitable alternative has the highest score. To calculate the final score for alternative Ai, the following equation is employed (Jadhav and Sonar 2009a, 2011):

32 $$S\left( {A_{i} } \right) = \sum W_{j} S_{ij}$$

where W_j_ is the importance value of the jth criterion; S_ij_ is the score that measures how well alternative A_i_ performs on criterion C_j_.

According to Refs. Zaidan et al. (2015), Jadhav and Sonar (2011), Triantaphyllou and Lin (1996), Whaiduzzaman et al. (2014), the characteristics of the above MCDM techniques can be summarized as follows. The WSM technique is easy to use and understandable. However, the weights of the attribute are assigned arbitrarily; thus, the task becomes difficult when the number of criteria is high. In Refs. Silva et al. (2013), Jadhav and Sonar (2009a, b), the AHP approach was utilized for software selection because it is a flexible and powerful tool for handling both qualitative and quantitative multi-criteria problems. Furthermore, AHP procedures are applicable to individual and group decision making. However, AHP is time consuming because the mathematical calculations and number of pairwise comparisons increase with the increasing number of alternatives and criteria. Another problem is that decision makers need to re-evaluate alternatives when the number of criteria or alternatives changes. However, ranking the alternatives depends on the alternatives considered for evaluation. Thus, adding or deleting alternatives can change the final rank (rank reversal problem). The ELECTRE technique can handle both qualitative and quantitative criteria. This technique provides a basis for a complete order of different options. The VIKOR technique uses linear normalization. However, the values are not dependent on just criterion evaluation but also on an aggregate function to balance the distance between both the ideal solution and its opposite. TOPSIS is functionally associated with the problems of discrete alternatives. It is one of most practical techniques for solving real-world problems. The relative advantage of TOPSIS is its ability to identify the best alternative quickly. The major weakness of TOPSIS is that it does not provide weight elicitation and consistency checking for judgments. From this viewpoint, TOPSIS meets the requirement of paired comparisons, and the capacity limitation may not significantly dominate the process. Hence, this method would be suitable for cases with a large number of criteria and alternatives, particularly for objective or quantitative data. In a fuzzy-based approach, decision makers can use linguistic terms to evaluate alternatives that improve the decision-making procedure by accommodating the vagueness and ambiguity in human decision making. However, computing fuzzy appropriateness index values and ranking values for all alternatives are difficult.

The limitation of the study in this report is multifaceted, we covered the subject by reviewing technical literature. We recognized numerous limitations in our study. First, the work in this paper applies only to the OSS-LMSs found on search engine databases. The list was selected in January 2014 by using several databases including ScienceDirect, IEEE Xplore, and Web of Science. The keywords used in the search included “open source software”/“learning management system” or “open source software”/“e-learning system” among others. The list of included software is not comprehensive but represents current active and popular projects at the time of study to support a manageable and valid software sample. Second, in an open source world, considerable change could be expected in the span of one-and-a-half years, including the rise and fall of projects. Moreover, more studies are required to identify the current evaluation criteria because many OSS-LMSs may be updated and/or added over the coming years.

There are some contributions in this paper listed as the following:

- Outlined samples of selection and active OSS-LMS packages with brief description in education
- Specified the criteria to evaluate OSS-LMS packages based on two aspects; identified and established then a crossover between them to highlight the gaps in the evaluation criteria used for OSS-LMS packages and selection problems.
- Discussed the ability of MADM/MCDM methods as a recommended solution in the future that is suitable to solve the problem of OSS-LMS packages in multi-criteria evaluation and selection problem and select the best OSS-LMS packages.

## Conclusions

Several aspects related to the OSS-LMS evaluation and selection were explored and investigated. In this paper, comprehensive insights are discussed on the basis of the following directions: ascertain available OSS-LMSs from published papers; specify the criteria of evaluating OSS-LMS packages on the basis of two aspects; identify and establish a crossover between them to highlight the gaps in the evaluation criteria used for OSS-LMS packages and selection problems. The ability of selection methods that are appropriate for solving the problem of OSS-LMS packages on multi-criteria evaluation and selection problem is discussed to select the best OSS-LMS packages. The outcomes from these directions are presented in list of active OSS-LMSs consisting of 23 systems. The open issues and challenges for evaluation and selection are highlighted. Other research directions include coverage and MADM/MCDM techniques that are related to the recommended solutions, which can be discussed on the basis of researchers’ opinion of the problem design and adoption of each technique. This research direction is significant because it will help administrators and decision makers in the field of education to select the most suitable and appropriate open source LMS for their needs.
